# CPBR Process in Urban Settings and the Unique Challenges and Successes

**DOI:** 10.1093/geroni/igab046.899

**Published:** 2021-12-17

**Authors:** Steffi Kim

**Affiliations:** University of Alaska Anchorage, Anchorage, Alaska, United States

## Abstract

CBPR is a framework that allows for the collaboration of researchers and communities as co-partners and is a supported approach for Indigenous communities. The community engagement and co-partnership in this study allowed for the researcher's flexibility to be responsive to culturally appropriate practices and priorities of the communities and participants. CBPR principles, including the Elder Advisory Committee (EAC), were utilized in this urban-based project. Challenges presented in many ways, including the processes of a) entering communities, b) relationship building, c) time involvement, and d) recruitment. Successes represented the unique opportunity to enter communities at an interpersonal level, b) close community engagement, c) gathering information beneficial for the research team and the community, and d) extended community engagement. While challenges exist, this approach's benefits are far-reaching promoting trust, support, and interest in future research endeavors. The presenter will discuss strategies and processes helpful in engagement, recruitment, and data collection.

